# Slither Into the Heart: Salmonella Endocarditis Following Rattlesnake Meat Ingestion

**DOI:** 10.7759/cureus.16466

**Published:** 2021-07-18

**Authors:** Kunal Mishra, Cameron Cu, Mehran Abolbashari, Chandra P Ojha, Jorge L Cervantes, Haider Alkhateeb

**Affiliations:** 1 Internal Medicine, Texas Tech University Health Sciences Center, El Paso, USA; 2 Cardiovascular Medicine, Texas Tech University Health Sciences Center, El Paso, USA; 3 Microbiology, Texas Tech University Health Sciences Center, El Paso, USA

**Keywords:** infective endocarditis, salmonella infection, rattlesnake meat, salmonella infective endocarditis, us-mexico border, cultural practices

## Abstract

Salmonellae foodborne infections are a well described and documented entity, however cardiac complications of Salmonellae foodborne infections including infective endocarditis (IE) are rare. Here we present a case of infective endocarditis as a result of bacteremia caused by multiple species of *Salmonella*. The patient initially presented with chest pain, fever and altered mental status. Troponin and ECG were unremarkable. The patient was started on empiric antibiotics. Blood cultures grew *Salmonella* species serotype O&H. Transesophageal echocardiogram (TEE) confirmed aortic valve vegetation. Regional cultural practices suggested possible contamination attributed to ingestion of rattlesnake meat, a practice that has been previously described and well-established in various Hispanic folk practices. Upon further history taking, the patient was found to be regularly consuming dried rattlesnake meat preparations, a rather common practice in Chihuahua desert region. Surgery was not indicated, and the patient was treated with six weeks of antibiotics. This case presents an opportunity to gain insight into such a unique manifestation of Salmonellae, offering a potential facet of information for clinicians to better understand its presentation, susceptibility, and potential adverse outcomes.

## Introduction

Infective endocarditis (IE), a once rare disorder, has been increasing in incidence in the US with the Midwest having the highest increase in rate [1]. Per a recent study, between the year 2000 and 2011 the incidence of IE increased from 11 per 100,000 to 15 per 100,000 [2,3]. Common risk factors that may predispose to the development of IE include the male sex, intravenous (IV) drug abuse, age >60, poor dentition, pre-existing structural heart or valvular disease, chronic hemodialysis, and HIV [4]. The precise reason for this rise in incidence is difficult to ascertain, as the risk factors and etiologies for developing IE are vast and vary geographically. These diverse risk factors also impact the distribution of healthcare-associated IE vs. community associated. While hemodialysis catheters, invasive procedures, prosthetic valves and implantable cardiac devices impact the incidence of healthcare-associated IE, IV drug abuse alone accounts for the significant rise in proportion of community-associated cases [4]. In fact, while IV drug abuse-related IE has been the reason for the fast rise in community-associated IE nationally, of all regions, the Midwest in particular has been heavily impacted, having the greatest increase in these cases [1].

The etiology of IE also is diverse, with a variety of micro-organisms that may lead to it [4,5]. The three most common organisms implicated in the etiology of IE worldwide are Staphylococci, Enterococci and Streptococci, with *Staphylococcus aureus* being the most common cause of IE in the US [4]. The HACEK (*Haemophilus species, Aggregatibacter actinomycetemcomitans, Cardiobacterium hominis, Eikenella corrodens, Kingella species*) organisms are a group of well-known fastidious gram-negative bacteria that also can cause IE [4]. While prognosis varies based on specific patient characteristics such as age or comorbidities, complications such as heart failure or septic shock, echocardiographic findings such as abscess or valve destruction, the management and prognosis of these patients is well documented [4,5].

*Salmonella* species remains a widely unusual cause of infective endocarditis with previous studies showing less than 3% of cases of bacterial endocarditis being attributed to Salmonellae which reflects the rarity of cardiac involvement by this genus [6]. Due to limited research, the clinical features and optimal treatment on *Salmonella* endocarditis remain unclear. However, per a recent review from 1976 to 2014, the prognosis of 87 reported cases was grave with a mortality rate of 42.5%. The source of Salmonellae can be from contaminated food and water or acquired via fecal-oral route from other humans or pets [6]. Rattlesnake-based products however represent a rather uncommon source of *Salmonella* [7]. A number of previous studies have identified various exposures specifically tied to ethnic groups with particular folk-medicine and culinary practices. Such knowledge was ultimately used in this case which yielded the origin of *Salmonella* infection [7,8].

This article was previously presented as a poster and abstract at the American College of Cardiology with World Congress of Cardiology (ACC.20/WCC) conference in March 2020.

## Case presentation

**Figure 1:**
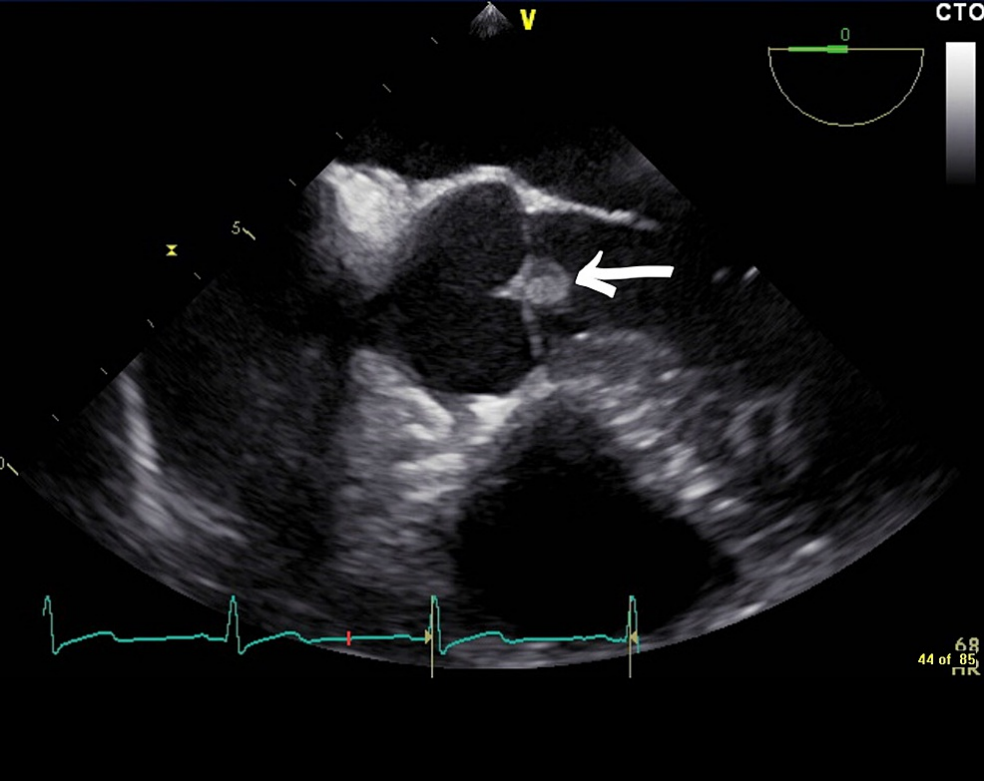
Mid Esophageal Long Axis View at Zero Degrees of Aortic Valve Vegetation.

A 50-year-old Hispanic male presented with moderate chest pain and discomfort that began the night prior to admission. On presentation, the patient was noted to have a fever of 39.5°C and found to be tremulous with marked altered mental status. Shortly after admission, the patient reportedly suffered multiple witnessed generalized tonic-clonic seizures for which he was emergently treated with lorazepam. The rest of the history could not be obtained from the patient due to his somnolent status. The family was therefore called at the patient’s bedside. They reported that the patient has a history of heavy alcohol consumption and was complaining of chest pain prior to presentation. They denied any history of seizures or patient noting any weakness or numbness prior to arriving to the hospital. Given the patient’s history of alcohol withdrawal, persistent fever and deteriorating clinical condition, the patient was taken to medical ICU for treatment of alcohol withdrawal. The patient did not have any other known chronic medical conditions. Initial complete blood count showed leukocytosis of 19.63 with bandemia and metabolic profile showed lactic acidosis. Initial electrocardiogram evaluation demonstrated sinus tachycardia with an unremarkable cardiac physical examination. Initial chest X-ray was unremarkable and non-enhanced CT of the head did not show any acute intracranial abnormalities. Ultrasound of the liver showed findings of hepatic steatosis, with patient having negative results for hepatitis panel, human immunodeficiency virus (HIV) and anti-nuclear antibody (ANA). The patient met systemic inflammatory response syndrome (SIRS) criteria with a heart rate of 108 beats per minute, fever of 39.5°C and leukocytosis of 19.63 for which reason two sets of blood cultures from different sites were collected for microbiological analysis and the patient was started on empiric piperacillin-tazobactam within hours of admission. Soon after initiating treatment for sepsis and alcohol withdrawal, patient’s altered mental status resolved, and his condition began to improve. Subsequent neurological exams done on the patient were all unremarkable with no signs of focal neurological deficits. Blood cultures subsequently grew multiple gram-negative rod species, later confirmed as various Salmonella spp. on two out of two plates at 12 and 24 hours, respectively. Antibody analyses revealed the following serotypes: Salmonella H, type-a, Salmonella H, type-b, Salmonella H, type-d, and Salmonella O, type-Vi. These findings prompted a transthoracic echocardiogram study which was unremarkable which led to a subsequent transesophageal echocardiogram (TEE). The TEE ultimately revealed a small 2.5 mm x 2.7 mm echo dense vegetation or mass observed on the aortic valve (Figure 1).

Given the patient’s aortic native valve endocarditis and gram-negative non-HACEK etiology per the IDSA/AHA guidelines, the patient as started on dual therapy, which based on susceptibility resulted in a regimen of cefepime and levofloxacin. As per the ACC/AHA guidelines, the patient did not meet the criteria for surgical considerations. The patient did not meet the criteria of complicated IE, which included findings such as heart failure, intra-cardiac abscess or fistula, native valve *S. aureus* IE, or systemic embolization. Hence cardio-thoracic surgery input was not sought and prolonged antibiotic therapy was recommended [9]. After multiple negative blood cultures at 48 hours, along with clinical stabilization at the hospital the patient was discharged on oral levofloxacin 750 mg and IV cefepime 2 g twice daily for six weeks. At his six-week follow-up, the patient was asymptomatic, with negative blood culture and unremarkable labs at the end of therapy.

## Discussion

The etiology behind infective endocarditis is easily made in the presence of bacteremia with gram-positive cocci. Even HACEK organisms which once were troublesome to grow, can be diagnosed easily with contemporary blood culture systems which grow HACEK bacteria within the first five days of routine blood cultures [10]. However, blood cultures may also contain bacterium rarely associated with endocarditis such as *Klebsiella spp*.,* Campylobacter* and even some fungi such as *Candida* or *Aspergillus* [4,11]. *Salmonella* species however represents a very rare etiology of IE.

The first case of *Salmonella* spp. endocarditis was reported in 1967, and a total of 87 cases have been reported from 1976 to 2014 [6,11]. Salmonellae are classified as flagellated Gram-negative facultative anaerobic Enterobacteriae that contain a broad range of clinically-relevant infective subtypes [12]. Salmonellae are typically spread through food and water contaminated with feces, which commonly cause systemic illness with or without associated diarrhea [11]. Beyond gastroenteritis symptoms, Salmonellae have been known to cause enteric fever, endovascular infections, osteomyelitis or present as an asymptomatic chronic carrier state.

*Salmonella* species have a proclivity to infect cardiovascular structures in adults. They may adhere to a damaged endothelium, predisposing individuals to complications rarely seen with other gram-negative organisms. *Salmonella* species make use of differing epithelial cell receptors for translocation into the gastrointestinal mucosa [12]. However, it is less clear how *Salmonella* species infect endothelial lined vessels. Various invasion factors allow invasion of endothelial cells, while also promoting being shuttled by macrophages after phagocytosis of the bacteria [12,13].

*Salmonella enterica* subsp. enterica serotypes are divided into typhoidal and nontyphoidal with enteric fever being caused by serotype Typhi and paratyphi and the other serotypes being collectively known as nontyphoidal [14]. These can be further subdivided based on serogrouping using antisera directed against a specific lipopolysaccharide (O) and flagellar (H) antigens. In our particular case our cultures identified four subtypes of Salmonellae: Non-typhi groups included *Salmonella* serogroup D with H antigen, *Salmonella* serogroup B with H antigen and *Salmonella* type Vi, which could be Typhi or Paratyphi [9]. It is unclear if the etiology of this IE case is due to the presence of multiple serotypes. Although many serotypes have been implicated, the majority of cases are caused by *S choleraesuis*, *S typhimurium*, and *S enteritidis* [9]. Furthermore, literature review supports both typhoid and nontyphoid *Salmonella* being associated with IE [6].

Regardless of the type of *Salmonella*, management of bacterial endocarditis remains the same. Surgery may be indicated if patients have signs of complicated IE which involve valvular dysfunction resulting in heart failure, development of an abscess, recurrent embolic events, prosthetic valve infection, *S. aureus* etiology or persistent bacteremia [9,15]. As our patient did not meet any of those criteria cardiothoracic surgery was never consulted. Optimal medical therapy of infective endocarditis requires prolonged antibiotic period. Per IDSA and AHA criteria for gram-negative non-HACEK endocarditis, the patient was placed on dual therapy of beta lactam (Cefepime in our case) and a fluoroquinolone (Levaquin in our case) for six weeks [9].

While management may not change with the subspecies or serotype of *Salmonella enterica*, this information can sometimes aid in narrowing down the source of infection. However, given that our patient had denied any contact with feces-contaminated food or water with no obvious source of infection we started looking for other sources of infection. The largest case series to date on endovascular infections related to Salmonella noted that HIV, cancer, cirrhosis and lupus increased the risk of bacteremia. While our patient did have history of alcohol abuse, he was noted to have hepatic steatosis and he was also found to be HIV and ANA negative. The negative immunodeficiency workup was particularly important here as case reports have found that patients who are immunodeficient, particularly those with acquired immunodeficiency syndrome (AIDS), are at higher risk of *Salmonella* endocarditis with the majority of case reports showing immunodeficient patients more likely to fall victim to this disease [16,17].

Given that our patient did not have any of the common risk factors, further history taking was done, after which the patient was found to be regularly consuming dried rattlesnake meat preparations, a rather common practice in the Chihuahua desert region. Literature review showed that up to 21 *Salmonella* spp. subtypes have been previously isolated from rattlesnake preparations, thus it may have been inevitable that our patient presented with salmonellosis given the frequency of his rattlesnake meat consumption [7,18]. Other studies have identified rattlesnake meats as a source of *Salmonella arizonae* infection [7,8]. Furthermore, rattlesnake capsules which are often marketed as remedies for certain conditions such as HIV infection or cancer or given by community “healers”, have been shown to be associated with Salmonella infections [19]. No research has supported any benefit from ingestion of rattlesnake capsules in the treatment of those diseases [7,8,17].

Patients affected by such rare infections are oftentimes traced to specific cultural beliefs and local culinary practices that involve the ingestion of either dried-rattlesnake meat preparations, or the consumption of rattlesnake capsules as a natural remedy [7,8,16,17]. This case presents an opportunity for physicians to recognize rare sources of IE by looking deep into cultural exposures and practices. By taking a detailed patient history, with careful attention to eating habits and ingestion of other supplements that can potentially explain a large portion of such unusual causes of bacteremia and the majority of associated, potentially fatal complications (Table 1).

**Table 1:** Features specific to Salmonella infective endocarditis secondary to rattlesnake ingestion

*Salmonella* Endocarditis secondary to rattlesnake etiology features	
When to suspect rattlesnake etiology for *Salmonella *infection	For diseases such as AIDS, diabetes, arthritis and cancer, ask patients if they have been seeking alternative forms of treatment such as rattlesnake capsules. For Hispanic patient populations from Tijuana or Juarez, Mexico, Los Angeles, California, various southern cities in Arizona, ask if any ingestion of rattlesnake meat has occurred.
Therapeutic differences in *Salmonella* endocarditis patients	Per IDSA and AHA criteria for gram-negative non-HACEK endocarditis, patient was placed on dual therapy of beta lactam and a fluoroquinolone for six weeks. Therapy is the same regardless of the type of *Salmonella*.
Valves impacted by *Salmonella*	Commonly impacts the mitral or aortic valve and may also infect cardiac walls (i.e. mural endocarditis).

## Conclusions

When approaching infective endocarditis patients, one must also consider rare etiologies that may have increased prevalence in certain geographical locations. Salmonellae is a rare etiology of endocarditis with potential deadly complications if not caught early. While cases in literature have shown immunocompromised patients to be at most risk of *Salmonella* IE, the patient seen in this case had no such conditions. It was only with more history taking that we discovered rattle-snake meat ingestion to be the culprit. This case showcases how in addition to a good history, clinicians must look beyond obvious risk factors and be aware of cultural practices that may explain the etiology of bacterial infections.
